# Overcoming Barriers to Mobilizing Collective Intelligence in Research: Qualitative Study of Researchers With Experience of Collective Intelligence

**DOI:** 10.2196/13792

**Published:** 2019-07-02

**Authors:** Van Thu Nguyen, Bridget Young, Philippe Ravaud, Nivantha Naidoo, Mehdi Benchoufi, Isabelle Boutron

**Affiliations:** 1 INSERM U1153 Epidemiology and Biostatistics Sorbonne Paris Cité Research Center (CRESS) Methods of Therapeutic Evaluation of Chronic Diseases Team (METHODS) Paris France; 2 University Paris Descartes Paris France; 3 Department of Health Services Research Institute of Population Health Sciences University of Liverpool Liverpool United Kingdom; 4 Centre d’Epidémiologie Clinique Hôpital Hôtel Dieu Assistance Publique des Hôpitaux de Paris Paris France

**Keywords:** collective intelligence, crowdsourcing, open innovation, health, research, survey, interview

## Abstract

**Background:**

Innovative ways of planning and conducting research have emerged recently, based on the concept of collective intelligence. Collective intelligence is defined as shared intelligence emerging when people are mobilized within or outside an organization to work on a specific task that could result in more innovative outcomes than those when individuals work alone. Crowdsourcing is defined as “the act of taking a job traditionally performed by a designated agent and outsourcing it to an undefined, generally large group of people in the form of an open call.”

**Objective:**

This qualitative study aimed to identify the barriers to mobilizing collective intelligence and ways to overcome these barriers and provide good practice advice for planning and conducting collective intelligence projects across different research disciplines.

**Methods:**

We conducted a multinational online open-ended question survey and semistructured audio-recorded interviews with a purposive sample of researchers who had experience in running collective intelligence projects. The questionnaires had an interactive component, enabling respondents to rate and comment on the advice of their fellow respondents. Data were analyzed thematically, drawing on the framework method.

**Results:**

A total of 82 respondents from various research fields participated in the survey (n=65) or interview (n=17). The main barriers identified were the lack of evidence-based guidelines for implementing collective intelligence, complexity in recruiting and engaging the community, and difficulties in disseminating the results of collective intelligence projects. We drew on respondents’ experience to provide tips and good practice advice for governance, planning, and conducting collective intelligence projects. Respondents particularly suggested establishing a diverse coordination team to plan and manage collective intelligence projects and setting up common rules of governance for participants in projects. In project planning, respondents provided advice on identifying research problems that could be answered by collective intelligence and identifying communities of participants. They shared tips on preparing the task and interface and organizing communication activities to recruit and engage participants.

**Conclusions:**

Mobilizing collective intelligence through crowdsourcing is an innovative method to increase research efficiency, although there are several barriers to its implementation. We present good practice advice from researchers with experience of collective intelligence across different disciplines to overcome barriers to mobilizing collective intelligence.

## Introduction

Innovative ways of conducting research have emerged recently with promising results. For example, Harvard Medical School organized an ideas competition, which attracted participants from 17 countries who contributed 150 new research ideas for managing type 1 diabetes [1]. In China, a creative competition involving participants from diverse backgrounds such as graphic designers, artists, and students resulted in new interventions to increase the HIV testing rate [2]. These initiatives were based on methods of mobilizing collective intelligence through crowdsourcing [3,4]. Collective intelligence is defined as shared intelligence emerging when people are mobilized within or outside an organization to work on a specific task that could result in more innovative outcomes than those when individuals work alone [5]. Crowdsourcing is “the act of taking a job traditionally performed by a designated agent and outsourcing it to an undefined, generally large group of people in the form of an open call” [6]. Although collective intelligence can emerge from day-to-day collaboration in science, by crowdsourcing, a large number of individuals with diverse backgrounds and expertise are enabled to contribute to research, resulting in collective intelligence on a large scale [3].

Use of such methods is increasing markedly across different disciplines. From 2010 to 2018, the number of projects mobilizing collective intelligence hosted by the US government through the website www.challenge.gov increased by more than 250% [7]. Collective intelligence enables researchers to solve problems, generate new research ideas, create intellectual products, and critically appraise research ideas and work [8-15]. For example, an initiative called Transparence Science created an online community of physicians and patients to develop a clinical trial protocol together [16].

Some resources describing methods of mobilizing collective intelligence in health research have been published [17,18]. However, literature on barriers that researchers encounter across different disciplines when mobilizing collective intelligence, advice on how to overcome these barriers, and good practice in mobilizing collective intelligence is still lacking. Our study aimed to identify the barriers to mobilizing collective intelligence and ways to overcome these barriers and provide good practice advice for those planning and conducting collective intelligence projects across different disciplines.

## Methods

### Study Design

To investigate collective intelligence methods, we conducted (1) a multinational online open-ended survey that allowed us to access the perspectives of a diverse group of respondents involved in collective intelligence and (2) semistructured interviews that allowed for more in-depth exploration of respondents’ perspectives on this fairly new topic.

Our approach was pragmatic when providing insights on the methods of mobilizing collective intelligence, but interpretive when analyzing respondents’ reports as subjective accounts of their experience when using these methods. The study received ethical approval (Ref: 17-386) from French National Institute of Health and Medical Research Ethic Committee (IRB00003888).

### Reflexivity

We have extensive experience in clinical trial methodology and an interest in understanding the method of mobilizing collective intelligence through crowdsourcing to apply it in clinical research. Some members of the team have conducted projects mobilizing collective intelligence.

### Sample and Recruitment

We recruited principal investigators and project coordinators experienced in running collective intelligence projects. We purposively sampled these researchers, seeking diversity in terms of their experience of different collective intelligence methods and their disciplinary backgrounds. We identified authors of articles reporting a project using collective intelligence [8], included researchers in the network of European citizen science association [19], and invited speakers in collective intelligence conferences [20,21]. We also used snowball sampling, asking respondents to send us email addresses of colleagues active in the field of collective intelligence.

An invitation email was sent via Mailjet [22] to researchers and project coordinators whose email addresses were available. The invitation contained a link to the first page of the survey, through which they indicated their consent. Two reminder emails were sent to nonrespondents.

We proposed semistructured interviews to a purposive sample of 24 researchers who did not respond to the first email invitation and who were mainly using collective intelligence in biomedical research and citizen science projects. They were invited via a personalized email sent by VN.

### Online Open-Ended Survey

The survey was developed using the results of a scoping review [8] and then pilot tested (Multimedia Appendix 1). It comprised five closed-ended questions to identify respondents’ background and expertise, and four open-ended questions exploring their motivation and experience with mobilizing collective intelligence, particularly the barriers they encountered and their solutions (Textbox 1). Finally, respondents were asked to provide three pieces of advice to a colleague planning to use collective intelligence in a project for the first time. To promote interaction between participants, we also asked them to rate and comment on the advice that other respondents had entered; the advice shown to each respondent was randomly selected from the pool of advice provided by previous respondents.

**Open-ended questions in the online survey.**

- What are *the benefits of collective intelligence* that made you decide to use it in your project?
- What were the *most important factors* contributing to the success of mobilizing collective intelligence in your project?
- What were the *most challenging issues* you had to face when using collective intelligence in your project and *your solutions for those challenges* (eg, difficulties in identifying and motivating participants, designing tasks for participants, evaluate quality of participants’ contribution, decision making)?
- What *three pieces of advice* would you give to a colleague who intends to use collective intelligence in a project for the first time?
- Please read the advice from another participant. (Showing an answer from another participant). What do you think of this advice?

### Semistructured Interviews

We sent individuals who expressed an interest in being interviewed an information sheet about the study. Interviews were conducted according to participants’ convenience (eg, face-to-face, telephone, and teleconference [gotomeeting.com]), and oral consent was obtained.

The interview guide covered content similar to that of the survey questionnaire (Multimedia Appendix 2). VN conducted all interviews in English. These were audio recorded, transcribed verbatim by a native English-speaking transcriber, and anonymized by VN. Interviews lasted between 22 minutes and 1 hour (median: 34 minutes). After each interview, VN wrote a summary of the interview to record the reflections on the interview and initial thoughts for the analysis.

### Analysis

Analysis of open-ended survey responses and interview transcripts was thematic, drawing on the framework analysis [23,24]. The analysis was partly deductive, with some aspects informed by the previous literature on collective intelligence, but also inductive to identify new themes and ensure that the analysis was grounded in the data. VN led the analysis. Two senior researchers BY and IB periodically reviewed transcripts and commented on the developing analysis.

Open codes and categories were developed by a constant comparative approach, reading and re-reading data and considering it in the context of other data from the same respondent and in the context of the wider dataset [25]. An initial framework of themes and subthemes was developed based on the first eight interview transcripts and then imported into NVivo to code the remaining transcripts and survey entries. The framework was further refined throughout the process of analysis.

Data saturation was examined by the theme accumulation curve that presented the number of distinct themes generated against a number of units of analysis used to generate those distinct themes (Multimedia Appendix 3) [26].

Respondents’ survey comments on the advice provided by other respondents were categorized as “agree” (ie, positive comments), “disagree” (ie, negative comments), and “neither agree nor disagree” (ie, neutral comment or did not directly comment on the idea in the answer). Two coders (VN and NN) independently assessed the content of each comment and discussed this to reach consensus. We received 129 pieces of advice: 100 advices were commented on by other respondents, and 28 were commented on twice, resulting in 128 comments. Most comments (98/128, 77%) agreed with the advice provided by respondents, and only 9% (12/128) disagreed. We summarized advice that commentators disagreed with in Multimedia Appendix 4.

The themes described below are derived from both interviews and survey entries. We present excerpts from the interviews and survey to explicate the findings and our interpretation of the data. Interviewees are indicated by “I” and survey respondents are indicated by “S”; “[…]” denotes text removed for brevity. Research disciplines of interviewees and survey respondents are listed in Multimedia Appendix 5.

### Data Sharing

The anonymized data from the online survey will be deposited on Zenodo, an open-access research data repository. Anonymized transcripts of interviews will be provided upon request.

## Results

### Respondent Characteristics

Of 157 people who clicked the survey link, 65 participated in the survey. Of the 24 people who were invited for interview, 17 participated in it. Of those who were not interviewed, two were unable to schedule an interview within the time frame of the study, two advised the interviewer to contact another team member responsible for the projects, two did not respond, and one was unable to be interviewed in English. Table 1 presents the demographic characteristics of survey respondents and interviewees. Survey participants were mainly from the field of computer science (43%), while interviewees were mainly involved in biomedicine and health care (59%). They mostly mobilized collective intelligence to solve research problems (70%) and generate new ideas (46%).

**Table 1:** Respondent demographics.

Demographic information		Survey respondents (N=65)^a^, n (%)	Interviewees (N=17), n (%)	Total (N=82), n (%)
**Age groups (years)**				
	20-29	4 (6)	0 (0)	4 (5)
	30-39	27 (42)	1 (6)	28 (34)
	40-49	19 (30)	11 (65)	30 (37)
	50-59	8 (12)	3 (18)	11 (13)
	≥60	4 (6)	2 (12)	6 (7)
**Location**				
	Europe	42 (65)	11 (65)	53 (65)
	North America	18 (28)	6 (35)	24 (29)
	Asia	2 (3)	0 (0)	2 (2)
**Research field^b^**				
	Computer science	28 (43)	2 (12)	30 (37)
	Biomedicine and health care	9 (14)	10 (59)	19 (23)
	Engineering and technology development	9 (14)	0 (0)	9 (11)
	Economics, commercial, and business development	7 (11)	2 (12)	9 (11)
	Education and information studies	7 (11)	0 (0)	7 (9)
	Environmental science	5 (8)	2 (12)	7 (9)
	Psychology and social science	5 (8)	0 (0)	5 (6)
	Laws, politics, and governance	4 (6)	1 (6)	5 (6)
	Other	10 (15)	0 (0)	10 (12)
**Purpose of using** **collective intelligence** **in their projects^**b**^**				
	Solve problems (ie, participants propose solutions to difficulties given by organizers)	44 (68)	13 (76)	57 (70)
	Generate ideas (ie, participants contribute to new ideas for research and development)	32 (49)	6 (35)	38 (46)
	Evaluate ideas (ie, participants evaluate the quality of the ideas/work)	23 (35)	1 (6)	24 (29)
	Create intellectual outputs (ie, participants create health education materials, clinical trial protocols, and prognostic models)	16 (25)	1 (6)	17 (21)
	Other	10 (15)	0 (0)	10 (12)

^a^Data for two persons are missing.

^b^Respondents selected more than one option.

### Researchers’ Motivations for Mobilizing Collective Intelligence

Participants reported trying the methods of collective intelligence as a new way of conducting research because traditional research methods no longer fitted their needs (Table 2). They commented that research questions were becoming very complex, unlikely to be solved within a single discipline and by traditional models of research, where each team working in relative isolation impeded research efficiency.

Respondents also noted the personal “pleasure” they derived from working “in teams with other people” (I10). Collective intelligence helped make research more enjoyable and helped them “to find some bridge, to…better understand each other, work closely together and this has some huge impact.” (I02)

### Barriers To Mobilizing Collective Intelligence

**Figure 1:**
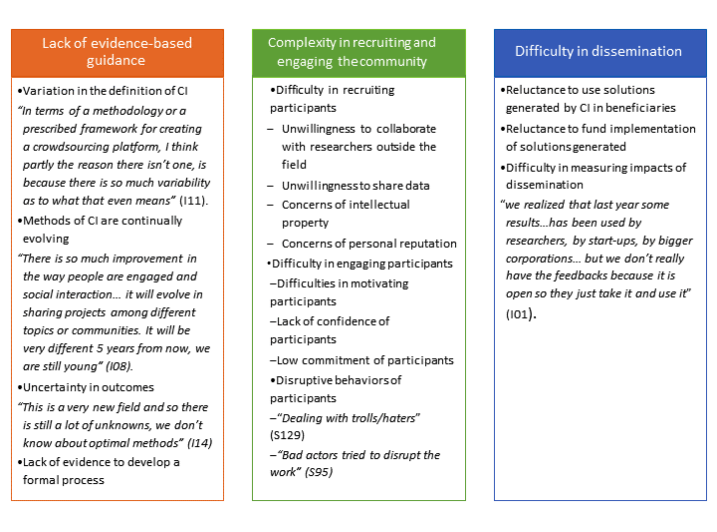
Barriers to mobilizing collective intelligence. CI: collective intelligence.

Although collective intelligence has numerous benefits, respondents found aspects of collective intelligence challenging. These challenges, in part, arose from the novelty of the method and complexity in engaging the community (Figure 1).

**Table 2:** Reasons for mobilizing collective intelligence.

Issues with traditional research practice	How collective intelligence can address the issue
Research questions were becoming more complex, and the answers could not be found from a single discipline	Collective intelligence provided the opportunity to work with people with different types of expertise and integrate their skills to solve problems from different angles: *Knowledge is distributed in different domains and some “wicked” questions cannot be answered within a single discipline or sector, ie, we need both different science disciplines as well as expertise from the practice and policy sector.* (S75)
Current research was conducted inefficiently by “repeating efforts” (I06)	Collective intelligence allowed researchers to conduct research as collective efforts where different approaches to a research question could be collectively and thoroughly evaluated to avoid redundant efforts: *In science, often we are developing solutions independently and we are kind of repeating erm…efforts, […] an alternative is to post a problem or a question to the research community and then just see what kind of solutions people come up with, and possibly combine these solutions and that you could call CI.* (I06)
As research questions became more complex, conducting research required a longer time. Researchers would not have enough time to investigate different aspects. “It takes for hundreds of years…you will never [be able to] explore everything.” (I08)	With a large community contributing, researchers were able to finish work within shorter time scales: *Draw on the experiences and expertise of a varied group of people to advance and implement ideas that would take a significantly longer time to solve as an individual.* (S104)
It was more costly to work with experts in the field and took longer to engage them	Mobilizing contribution from a wide community was cheaper than working with experts in the field, yet the former could achieve the same outcomes: *Our organization has done over 300 crowd-based challenges and has found success in 80-90% of those challenges with cost and schedule savings in the majority of them.* (S49)

#### Lack of Evidence-Based Guidelines on Methods of Mobilizing Collective Intelligence

Use of collective intelligence through crowdsourcing in research is relatively new. Some respondents reported that they had delved into this method before they had become fully aware of the concepts of collective intelligence, crowdsourcing, or citizen science. Respondents also recounted challenges they had faced in their projects due to lack of evidence for an “optimal method” (I14) and noted the absence of a methodological guide for them to follow.

#### Complexity in Recruiting and Engaging the Community of Participants

Respondents believed that some potential collective intelligence participants had “a lot of prejudice” (I03) toward collaborating with people from different fields, and it was “not easy to make them to participate” (I02) in collective intelligence projects. One interviewee (I06) working in the field of biomedicine spoke of the difficulties he experienced in motivating industrial partners to work with academic institutions in his challenge contests. He commented that collective intelligence participants had concerns about the ownership of the research intellectual property of solutions created and negative reputation consequences if their solutions performed poorly.

Respondents described difficulties in “retaining all the people that sign up…to get them to actually participate” (I09), as most participants joined collective intelligence as a side project or “an unfunded kind of project” (I12). They also believed that many potential collective intelligence participants were “not confident enough” (I07), which hindered their contribution.

Respondents reported situations when participants had tried to cheat or behaved aggressively, which adversely influenced the community and demotivated other participants. One interviewee shared his experience with this disruptive behavior, when organizing challenge contests for data analytics*:*

They will make different identities…and…submit hundreds [times]…[they] cheat the leader boards. [They] will discourage many people from [participating]…but [they]don’t have the solution.

I04

He explained that this disruptive behavior partly arose from the competitive nature of a contest, adding that participants might be under pressure from their organizations to win international contests in order to increase reputation of the organizations.

### Difficulties in Disseminating the Solutions Generated by Collective Intelligence

Respondents found it challenging to disseminate and implement the findings of their collective intelligence projects to the relevant communities, as funders and beneficiaries were unfamiliar with this emerging method. These challenges arose partly from the “prejudice” of researchers (I03) that people who were outside of the field might not have sufficient capacity to create solutions. One interviewee spoke of his difficulty in persuading funders to sponsor the further development of solutions generated by collective intelligence participants in a challenge contest that he had organized:

The third challenge…was getting people to recognize that these solutions existed and were available…there is a reluctance to use crowdsource and open source solutions like this.

I15

### Good Practice Advice for Planning and Conducting Collective Intelligence Projects

**Figure 2:**
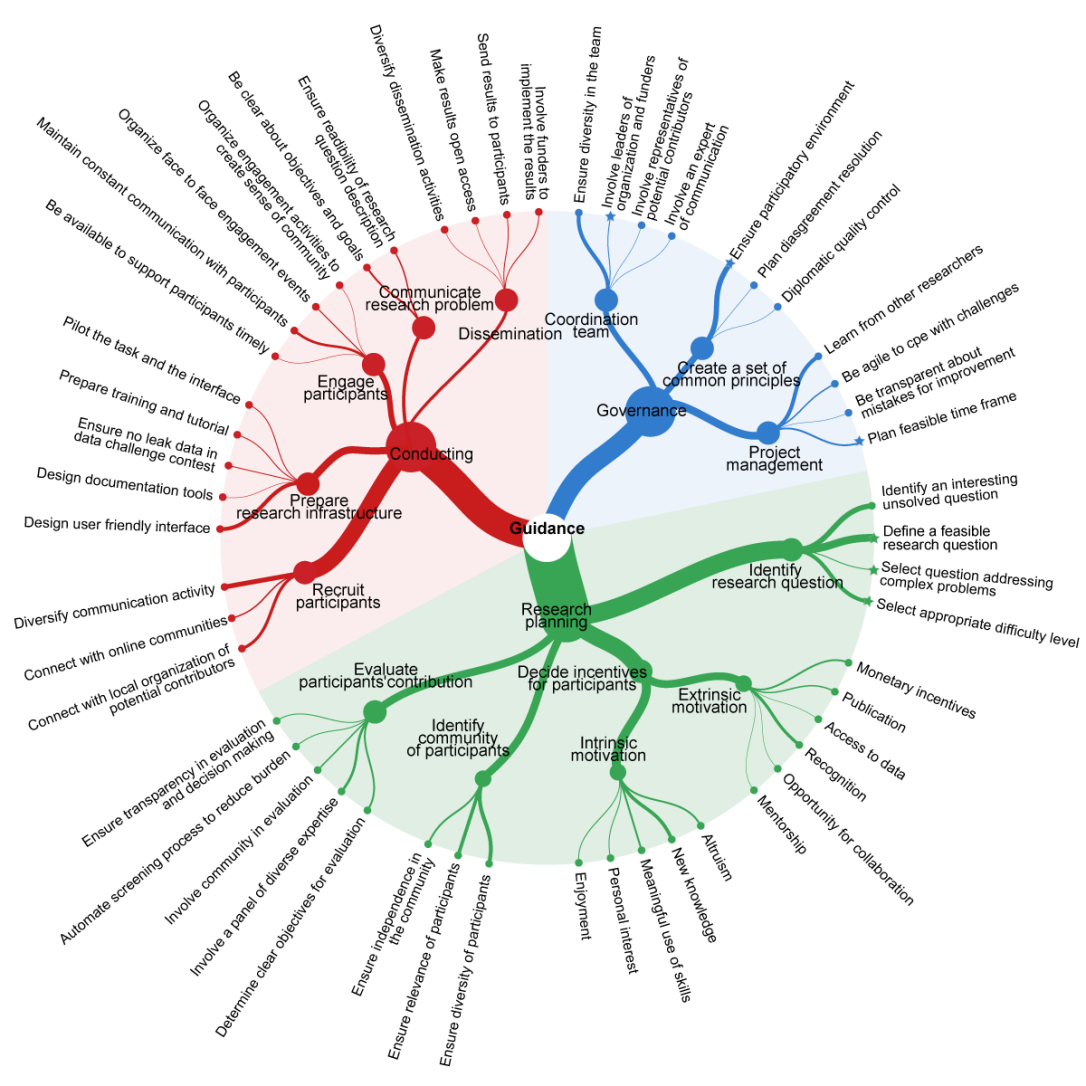
Good practice advice for planning and conducting research mobilizing collective intelligence.

When describing their projects, respondents reflected on the solutions that they had considered or used to overcome these barriers. We also explicitly sought their advice on what they perceived to be good practice in planning and conducting collective intelligence projects. In the sections that follow, we present respondents’ good practice recommendations for collective intelligence projects, covering three main themes: project governance, planning, and conduct of collective intelligence projects (Figure 2).

### The Project Governance

#### Establishing a Coordination Team

Respondents advised researchers to establish a coordination team dedicated to supporting projects mobilizing collective intelligence. They suggested that the coordination team should include people with diverse expertise to bring more “insights” (I01) to the project and help with “getting leadership and [funders] on board” (S23). Respondents also encouraged researchers to involve stakeholders and representatives from potential collective intelligence participant groups in planning, designing, and conducting collective intelligence projects.

Listen very carefully to your participants and work with them. Ensure mutual benefits in your design and co-create the project.

S62

Respondents advised that the involvement of participants’ representatives from early stage would help identify mutual research interests between participants and researchers, design appropriate tasks, and develop effective communication strategies to engage potential participants. Respondents also emphasized on the importance of including people with experience in communication in the team to support recruitment and engagement activities with collective intelligence participants.

#### Create a Set of Common Rules

Respondents suggested that the coordination team create a set of common rules for collective intelligence participants to encourage mutual respect and constructive contributions. They mentioned the use of “diplomatic quality control” (I03) to flag aggressive or disruptive behavior from participants and to try to create a participatory and friendly environment for others to freely contribute their work. They also suggested preparing a resolution plan to resolve conflicts between collective intelligence participants.

### Planning a Collective Intelligence Project

#### Identify the Research Question

Respondents commented that an early step in research involving collective intelligence was to identify “an interesting problem” (I06) with “high scientific value” (I04) that would gain from the involvement of a large and diverse community.

It is number one that there is a problem out there worth solving […], a project that it makes sense to try and bring in…people outside of the normal kind of scope or expertise area for it.

I15

They noted that identifying “just difficult enough” (I06) problems and “putting yourself in the participants’ [positions]” (I08) were crucial to create appropriate research problems to gain buy-in from target communities. One interviewee (I15) working in the field of biomedicine and health care described how a dynamic process involving “a lot of conversations” was part of the process of establishing whether the community would be interested in the research problem.

We knew there were a lot of people…working on it [the research topic] and no one had come up with an optimal solution and we felt like there were enough people who would be interested…but that didn’t come from us just sitting in a room alone. We actually reached out to many of the people…to see if they felt like there was a need and an ability to really take this further.

#### Identify Communities of Participants

Respondents also considered the choice of the communities a key factor in ensuring successful mobilizing of collective intelligence. Respondents suggested identifying communities who “have most contact with these problems” (I05).

You need to have champions of the cause…if you are doing something on Alzheimer’s, finding a person…who has Alzheimer’s, who their mother, father has Alzheimer’s and who has a personal vested interest and a strong…passion for the cause.

I14]

They emphasized on two important characteristics of the community—diversity and independence. Diversity in participants was thought to be important to generate novel solutions to the research problem. Diversity could be achieved by involving a larger number of participants with various disciplines.

The more participants you have, the more likely some of them will come with the new idea.

I04

Similarly, maintaining the independence of participants as they worked on the research problem was crucial to “free the minds and let [participants] think freely” (S104), allow “outside of the box thinking” (S146), and ensure that participants could voice their ideas without being influenced by a dominant opinion.

#### Decide on Incentives to Engage Participants

Respondents suggested offering a combination of both extrinsic motivators such as authorship and access to the data and intrinsic motivators such as making tasks enjoyable, offering participants the opportunity to gain new knowledge and finding meaningful outlets for their skills. They described some innovative activities to engage participants:

Some of the things that we have done have been really fun, and really brought the community together…to create…a sense of community…like the 24-hour citation screening challenges. Where we have had hundreds of people, online at the same time, all with a specific target to try and reach within 24 hours…and those have been hugely exciting, really popular.

I17

Interestingly, some respondents tried to “avoid monetary prizes” (I14), as they believed that “the crowd may only be interested in the compensation and therefore, may take short-cuts or cheat if the task allows for that” (S153). Instead, they suggested offering research partnership, mentorship, or training as ways to benefit participants’ professional development.

#### Determine Methods to Evaluate Solutions Created by Collective Intelligence and Decision Making

Respondents emphasized the need to “set up objective methods to validate the results” (S65), for example, by establishing a panel with diverse expertise to comprehensively evaluate contribution of participants. They also acknowledged the need to allow enough time for evaluation, given the large number of participants, and advised involving the crowd in the evaluation to increase the efficiency of the process. Automating screening of participants’ contributions was also suggested to reduce work load for the panel when performing the evaluation.

### Conducting Collective Intelligence Projects

#### Prepare Tasks and Interface

Respondents highlighted the need to design a user-friendly interface to “make it really easy for people to contribute even if they have only got a minute free” (I17). They explained that “the design of the interfaces or platforms which people will use is often overlooked but can influence the results or the ease of data collection” (S25).

They also advised researchers to prepare training materials and offer tutorials to explain the project to participants and equip them with essential skills. However, they noted that the training should avoid providing participants with examples that could hinder participants’ creativity.

Respondents also recommended “verifying if it [the task and interface] works on small scale” (S16) and gradually scaling up. The pilot phase could help researchers foresee any technical and ethical issues related to data collection and participants’ identities, which could be addressed before a large number of collective intelligence participants enrolled.

#### Create a Clear Description of the Research Problem

*Crafting* a clear description of the problem in a language relevant to those communities was considered a key step to helping collective intelligence participants understand the project objectives and judge whether they had the relevant skills to participate:

Good communication of a complex objective or complex data set…is not…always easy...if there is something that you don’t even understand,…you won’t put your time in that challenge

I10

One respondent also suggested dividing the objectives into concrete deliverables with clear requirements for participants’ contributions:

In order for the collective to provide “intelligence” as opposed to noise, one must be very careful about what one measures… If the measures are ambiguous to the participants, or if there exists a short-cut for the participants to satisfy immediate goal without actually contributing to the overall big picture, many participants will find this short-cut and will explore it

S20

#### Organize Communication Activities to Recruit Participants

Respondents described how they had organized various communication activities to recruit participants via advertisements on social media (eg, Google, Facebook, and other websites) and announcements in scientific publications. Several thought of working with an intermediary online platform, which had a readily available online community, as a practical approach for those who were new to collective intelligence. They advised researchers to partner with local organizations such as nongovernmental organizations, universities, and patient organizations and organize face-to-face meetings to connect directly with participants.

#### Engage Participants Through Responsive Communication

To engage participants effectively, respondents believed that communicating frequently with collective intelligence participants, even being available *24/7* to guide them and give feedback on their contributions. Respondents believed this would improve the quality of participants’ contributions and increase their commitment. Further, through responsive communication with participants, researchers could understand what resources participants needed for developing an implementable solution. Although virtual communication helped in ensuring responsive communication, respondents advised supplementing this with face-to-face engagement events to increase trust and create a sense of community among collective intelligence participants.

#### Disseminate Solutions Created by Collective Intelligence for Beneficiaries and Collective Intelligence Participants

Respondents advised researchers to diversify the dissemination of their project findings through multiple channels and make the results open access to the public through social media.

Respondents suggested involving leaders of organizations from the beginning of the projects to ensure their support for implementation of solutions generated by collective intelligence. They encouraged other researchers using collective intelligence to “show their results” (I02), “evaluate” (I13), and “be transparent about mistakes” (I17) and believed that rigorous evaluation of collective intelligence was necessary to provide evidence of its usefulness to stakeholders, “so that it gets recognised and funded properly” (I13).

## Discussion

Our study showed that researchers were interested in looking for efficient methods of conducting research, leading them to try collective intelligence. Researchers believed that by involving large numbers of participants with various disciplines, they could find more innovative solutions to research problems in a shorter time with fewer costs compared to conventional methods. They indicated that participants’ contributions could be solicited to solve problems, generate new research ideas, evaluate ideas, and create intellectual outputs. Researchers embarking on collective intelligence projects for the first time learned through the process and gradually improved their methods. They encountered barriers in planning and conducting collective intelligence projects due to the lack of a methodological guidance. We drew on the experiences of researchers across different fields and with experience of different collective intelligence methods to identify solutions and good practice advice to support researchers in the planning and implementation of their collective intelligence projects. This advice will help researchers prepare structures and processes for their projects, plan essential steps in their research, and foresee and develop strategies to overcome the barriers.

Despite increasing recognition of the value of collective intelligence in research [27,28], there are still examples of inappropriate methods used to mobilize collective intelligence [29]. For example, a project involving crowdsourcing in Rwanda failed to recruit and engage participants because the researchers mainly used social media for recruitment and requested participants to use a complicated tool for data collection [30]. However, community members in Rwanda were not connected on social media and were unfamiliar with the data collection tool. These issues could have been mitigated if the representatives of the target communities were involved from the outset as members of the project coordination team to advise on the conception and design of the collective intelligence project. A National Aeronautics and Space Administration competition to name a new node of the International Space Station was misled when an influential person called on the community to vote for his own name [31]. These examples emphasize the necessity of sharing experiences of researchers who have implemented collective intelligence projects to help future collective intelligence projects avoid methodological mistakes and outputs that are biased by group thinking.

Several efforts to define and standardize methods of collective intelligence in specific fields are available. These include a practical guide on using challenge contests to crowdsource ideas and solutions for health research from the World Health Organization and a list of toolkits compiled by the European Association of Citizen Science for researchers carrying out citizen science activities whereby members of the public collect and classify data [17,32]. However, a scoping review of the literature across different research fields classified four main methods to mobilize collective intelligence: independent contribution, challenge contest, games, and collaboration with a number of projects combining at least two methods [8]. By exploring experience of researchers who used one or more of these four methods in diverse disciplines, our study highlighted the barriers to mobilizing collective intelligence that researchers might encounter in different contexts. Good practice advice from researchers across disciplines could benefit researchers in planning and conducting future collective intelligence projects using one of these four methods within and outside health research.

To our knowledge, this is the first qualitative study to investigate the experiences of researchers in mobilizing collective intelligence across different fields. By using an online survey and semistructured interviews with a purposive sample of international researchers who had experience in implementing a range of different collective intelligence methods, we gained a breadth of perspectives. Respondents to the survey and interviews came from diverse disciplines, and some of them identified themselves as multidisciplinary researchers. The survey allowed a degree of interaction between researchers, which aided the analysis and interpretation of the results. Identification of areas that researchers agreed on helped us ascertain which barriers and strategies were applicable across different disciplines. Additionally, the semistructured interviews allowed researchers to explain the context of their research and describe their ideas and methods for addressing problems in mobilizing collective intelligence in depth.

Our study has some limitations. The online survey allowed participants to freely express their opinions, but we were unable to probe further to clarify the information written and gain a deeper understanding of their context. Furthermore, our survey and interview samples were mainly researchers who had published their collective intelligence projects. Therefore, we are uncertain about how far our findings are relevant to unpublished collective intelligence projects. Additionally, although we interviewed and surveyed researchers who had experience in running collective intelligence projects, we did not interview collective intelligence participants. Such data could provide further valuable insights on how to motivate and engage them.

In conclusion, mobilizing collective intelligence could be an effective way to improve research efficiency. The findings described in this paper should help researchers understand the barriers to implementing this new method. The good practice advice that we derived from respondents’ accounts aims to support researchers in mobilizing collective intelligence effectively.

## Appendix

Multimedia Appendix 1 Online survey questionnaire.

Multimedia Appendix 2 Interview guide.

Multimedia Appendix 3 Theme accumulation curve.

Multimedia Appendix 4 Advice that commentators disagreed with.

Multimedia Appendix 5 Respondents’ research disciplines.
