# Supplementary material for: Overcoming Barriers to Mobilizing Collective Intelligence in Research: Qualitative Study of Researchers With Experience of Collective Intelligence
Source: J Med Internet Res. 2019 Jul 2;21(7):e13792. doi: 10.2196/13792 (PMC6632103; doi:10.2196/13792)
Supplement: Multimedia Appendix 1 [file jmir_v21i7e13792_app1.pdf]

Appendix 1: Online survey questionnaire

Demographic information
1. What is your age range?<br>○ <20 ○ 40–49<br>○ 20–29 ○ 50–59<br>○ 30–39 ○ ≥60
2. Where are you located currently? (Dropdown list of continents)
3. What is your research field? (Please select all that apply)<br>☐ Biomedicine<br>☐ Psychology<br>☐ Technology development<br>☐ Computer science<br>☐ Education<br>☐ Laws, politics and governance<br>☐ Economics, commercial, business development<br>☐ Environmental science<br>☐ Other (please specify): ___________________
4. In how many projects have you used collective intelligence?<br>○ 1<br>○ 2–5<br>○ >5
Please refer to the most recent completed project in which you used collective intelligence and answer the following question
5. What is **the purpose of mobilizing collective intelligence** in your project?<br>☐ Evaluate ideas<br>☐ Generate ideas<br>☐ Solve problems<br>☐ Create intellectual products<br>☐ Other (please specify): __________________
6. What are **the benefits of collective intelligence** that aided your decision to use it in your project?
7. What were the **most important factors** contributing to the success of mobilizing collective intelligence in your project?
8. What were the **most challenging issues** you had to face when using collective intelligence in your project and **your solutions for those challenges** (e.g. difficulties in identifying and motivating participants, designing tasks for participants, evaluate quality of participants' contribution, decision making)?
9. What **three pieces of advice** would you give to a colleague who intends to use collective intelligence in a project for the first time?
10. Would you **use collective intelligence again**?<br>○ Definitely ○ Yes ○ Perhaps ○ No ○ Definitely

yes no<br>Please tell us why you choose that answer: ___________________________
11. Do you think collective intelligence will be **increasingly used in the future**?<br>o Definitely yes o Yes o Perhaps o No o Definitely no<br>Please tell us why you choose that answer: ___________________________
Please read the advice from another participant. (Showing an answer from another participant)<br>What do you think of this advice? Rate from 1 to 5 stars.<br>Please comment on this advice? (Free text box for writing comment)
