# Supplementary material for: Overcoming Barriers to Mobilizing Collective Intelligence in Research: Qualitative Study of Researchers With Experience of Collective Intelligence
Source: J Med Internet Res. 2019 Jul 2;21(7):e13792. doi: 10.2196/13792 (PMC6632103; doi:10.2196/13792)
Supplement: Multimedia Appendix 2 [file jmir_v21i7e13792_app2.pdf]

Appendix 2: Interview guide

Main topic	Questions
1. Background	• To start off, could you please tell me about yourself?<br>*Prompt*<br>- What is your area of research?<br>- When was the first time you heard about collective intelligence? How did you come up with the idea of using collective intelligence in your work? Do you work in a research group? What's your role in the group?<br>• Could you please share with me more about projects that you used collective intelligence?<br>*Prompt*<br>- How many projects have you used collective intelligence?<br>- What was your first project using collective intelligence? Your recent project?<br>• Taking one of your completed projects as an example, could you walk me through that project?<br>*Prompt*<br>- How did the initial idea come about? How did it get started?<br>- What were you and the team hoping to get out of using collective intelligence in your project?<br>- How did you and your team organize it?<br>*Prompt*<br>- Identify participants, motivations<br>- Tasks given to participants<br>- Evaluate contribution of participants and decision making
2. Facilitators to mobilize collective intelligence	• When looking back at projects that you used collective intelligence, would you say it was a success? In what ways?<br>• In your opinions, what were the factors contributing to the success of your project?<br>*Prompts*<br>- The community

	- The management team, expertise<br>- Interface of the platform<br>- Transparency in communication
3. Challenge in mobilizing collective intelligence	• What challenges did you face when using collective intelligence in your project?<br>**Prompt**<br>- Challenges in organization (establish core team, establish platform of organization, establish community)<br>- Challenges in identifying and engaging participants<br>- Challenges in designing tasks for participants<br>- Challenges in evaluating participants' contribution<br>- Challenges with data sharing and intellectual property<br>- Challenges in decision making<br><br>• Did you/your team overcome the challenges that you have mentioned? What did you do?
4. Future of collective intelligence	• What advice would you give to people who intend to use collective intelligence for the first time?<br>• Would you use collective intelligence again in your future projects? Please tell me more about that.<br>• Do you think collective intelligence will be increasingly used? Please tell me more about that. How do you think about the future of CI?<br>• Should we raise awareness of collective intelligence among researchers, funders and community? How could we do that?<br>• What do you think about the publication of methods of projects applying collective intelligence? What do you think about the dissemination of the results?<br>*Prompt*: Publication bias towards positive results, reproducibility of methods
5. Other	• Is there anything else that we haven't discussed that you would like to share?
