# Supplementary figures and images for: Overcoming Barriers to Mobilizing Collective Intelligence in Research: Qualitative Study of Researchers With Experience of Collective Intelligence
Source: J Med Internet Res. 2019 Jul 2;21(7):e13792. doi: 10.2196/13792 (PMC6632103; doi:10.2196/13792)

#### Appendix 4: Theme accumulation curve to assess data saturation

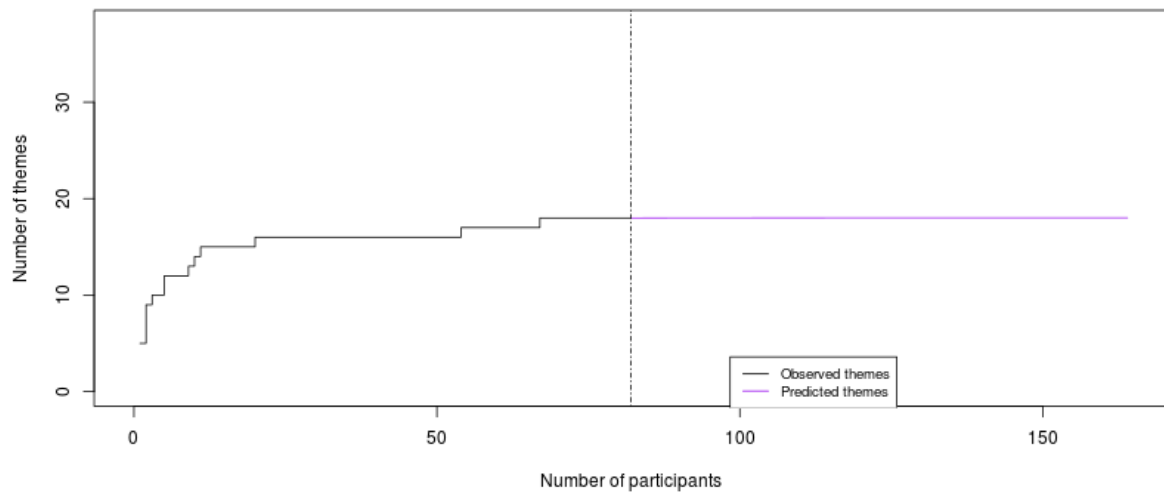

Supplement: Multimedia Appendix 3 [file jmir_v21i7e13792_app3.pdf]
