# Supplementary material for: Overcoming Barriers to Mobilizing Collective Intelligence in Research: Qualitative Study of Researchers With Experience of Collective Intelligence
Source: J Med Internet Res. 2019 Jul 2;21(7):e13792. doi: 10.2196/13792 (PMC6632103; doi:10.2196/13792)
Supplement: Multimedia Appendix 4 [file jmir_v21i7e13792_app4.pdf]

Appendix 5. Advice which commentators disagreed with

	Advice	Comment
Involve top leaders in organization	Planning is key. Make sure you get the CEO and leadership onboard, choose a question that can solve a big challenge	Agree but leadership is not important
Define feasible research questions	Be careful about goals and expectations, be ready to be flexible and adaptive, keep in mind what is your particular goal and be honest with all participants beforehand	Collective Intelligence can help refine a goal or redirect one that seemed good but turned out not to be.
Select appropriate difficulty level	Don't ask too much to the contributors, otherwise they won't participate (or won't finish their contribution)	Depends very much on what kind of data you are looking for, and what kind of crowd you are aiming at. Some amateurs of astronomy can follow elaborated protocols for decades. The only encouragement they need is channels through which they can submit their data and some sense of being acknowledged for their contributions to science. Members of the crowd in a more general sense, naturally needs way more encouragement, feedback etc.
Select questions address complex problems	Try to find the most complex challenge people can solve.	I would not necessarily go for the most complex challenge but an important and societally highly relevant challenge
Plan feasible time frame	Make studies short, since crowdsourced users have short attention span.	Mostly good, but studies don't have to be short. I've known projects that have been going for 10 years that over 30,000 people are still engaged with. If your project will take a long time, tell people that up front but let them know they can help as much or as little as they can.
Plan feasible time frame	Make studies short, since crowdsourced users have short attention span.	We're not only talking about mass crowdsourcing, but collective intelligence can also be used with a few experts, e.g. divers to map lake floors or archaeologically interested people to think about a problem etc. Some citizen science projects have run for a long time, but of course they do need to fit the time resources people have and be

		engaging and fun. Quality control is something we always do in science.
