# Supplementary material for: Overcoming Barriers to Mobilizing Collective Intelligence in Research: Qualitative Study of Researchers With Experience of Collective Intelligence
Source: J Med Internet Res. 2019 Jul 2;21(7):e13792. doi: 10.2196/13792 (PMC6632103; doi:10.2196/13792)
Supplement: Multimedia Appendix 5 [file jmir_v21i7e13792_app5.pdf]

Appendix 3: Research disciplines of survey respondents and interviewees.

Respondent identification number	Research disciplines
I01	Biomedicine and healthcare
I02	Open innovation
I03	Laws, politics, governance
I04	Computer science
I05	Economics, commercial, business development
I06	Biomedicine and healthcare
I07	Environmental science
I08	Environmental science
I09	Biomedicine and healthcare
I10	Computer science
I11	Biomedicine and healthcare
I12	Biomedicine and healthcare
I13	Biomedicine and healthcare
I14	Biomedicine and healthcare
I15	Biomedicine and healthcare
I16	Biomedicine and healthcare
I17	Biomedicine and healthcare
S01	Biomedicine and healthcare
S02	Biomedicine and healthcare, Computer science
S03	Information and communication
S04	Education
S05	Laws, politics, governance
S06	Biomedicine and healthcare
S07	Environmental science
S16	Computer science
S19	Economics, commercial, business development
S20	Computer science
S23	Education
S25	Computer science
S26	Computer science, Digital humanities
S31	Computer science; Economics, commercial, business development; Technology development
S32	Economics, commercial, business development
S33	Education
S34	Computer science; Economics, commercial, business development; Technology development
S39	Technology development
S40	Biomedicine and healthcare; Computer science
S42	Computer science; Education
S43	Economics, commercial, business development
S45	Education; Cheminformatics

S46	History
S47	Biomedicine and healthcare; Computer science
S49	Open innovation
S52	Biomedicine and healthcare; Computer science
S54	Biomedicine and healthcare; Computer science
S57	Computer science
S59	Computational linguistics
S62	Environmental science; Technology development
S65	Computer science
S66	Computer science
S67	Astrophysics
S70	Computer science; Economics, commercial, business development; Education; Technology development
S75	Environmental science
S83	No information
S86	Biomedicine and healthcare; Computer science
S88	Psychology
S92	Complex systems
S93	Computer science
S95	Emergency and disaster support
S96	Laws, politics, and governance
S100	No information
S101	Environmental science
S104	Technology development
S107	Laws, politics, governance
S109	Computer science; Economics, commercial, business development; Psychology; Technology development
S117	Social science
S120	Economics, commercial, business development
S122	Economics, commercial, business development
S123	Engineering
S128	Computer science
S129	Technology development
S130	Computer science
S133	Biomedicine and healthcare
S135	Citizen science
S141	No information
S142	Computer science
S143	Library archive
S146	Computer science
S149	Computer science; Economics, commercial, business development; Environmental science
S150	Computer science

S151	Computer science
S153	No information
S155	Computer science
